# Psychological functioning of patients with type I diabetes: the relevant role of alexithymia and intolerance to uncertainty

**DOI:** 10.3389/fpsyt.2025.1667103

**Published:** 2025-10-01

**Authors:** Emanuele Maria Merlo, Gabriella Martino, Rita Tutino, Liam Alexander MacKenzie Myles, Sean Hill, Angela Alibrandi, Maria Carmela Lia, Domenico Minasi

**Affiliations:** ^1^ Department of Biomedical and Dental Sciences and Morphofunctional Imaging, University of Messina, Messina, Italy; ^2^ Department of Clinical and Experimental Medicine, University of Messina, Messina, Italy; ^3^ Pediatric Unit of Ospedali Riuniti Presidium, Grande Ospedale Metropolitano Bianchi Melacrino Morelli, Reggio Calabria, Italy; ^4^ Department of Experimental Psychology, University of Oxford, Oxford, United Kingdom; ^5^ Department of Human and Pediatric Pathology ‘Gaetano Barresi’, University of Messina, Messina, Italy

**Keywords:** alexithymia, anxiety, clinical psychology, depression, diabetes, intolerance to uncertainty, psychopathology, type 1 diabetes mellitus

## Abstract

**Introduction:**

Type 1 Diabetes Mellitus (T1DM) represents a serious condition with high prevalence and increasing incidence. Phenomena such as alexithymia, intolerance to uncertainty and psychopathology are recognized as generally affecting individuals' health status and contributing to the worsening of T1DM. The present study aimed to study the relationships between these variables and differences relative to age and gender of T1DM patients.

**Methods:**

A sample of 150 participants aged between 11 and 18 years old with T1DM was recruited. All participants completed the diagnostic protocol, consisting of a sociodemographic questionnaire, Toronto Alexithymia Scale, Intolerance to Uncertainty Scale and Self Administrated Psychiatric Scales for Children and Adolescents (different psychopathological domains). Descriptive, correlational, regression and differential analyses were conducted.

**Results:**

Alexithymia and intolerance to uncertainty predicted psychopathology and significant differences were found among age and gender groups.

**Discussion:**

Despite this, more studies are necessary to improve knowledge in the field of psychological functioning in T1DM. Further research to deepen understanding of how alexithymia and intolerance of uncertainty influence psychological functioning in T1DM are indicated.

## 1 Introduction

Type 1 Diabetes Mellitus (T1DM) represents a serious chronic condition affecting one in three hundred individuals worldwide, with a growing incidence (1, 2). Recent research demonstrated the need for multifactorial approach to managing the different etiological factors affecting participants’ quality of life (3–9).

Psychological factors have been recognized to play a fundamental role in the management of chronic conditions (10–17). With particular reference to T1DM, psychological phenomena have gained attention in the last twenty years, with evidence indicating they impact the onset, maintenance and worsening of the disease (7, 18–24, 186, 187). Thus, it is clear that continuous negligence of these factors can constitute a serious risk factor, affecting the physical and psychological health of patients. Several studies have considered the role of relevant variables such as age, gender and education. Despite these sociodemographic variables remain fundamental for research (25–30), including psychological variables in the study of T1DM dynamics represents important progression. For instance, recent studies demonstrate that the need of screening for risk factors linked to T1DM is fundamental (31). In fact, particular circumstances linked to gender, age, duration of the disease and psychopathological conditions have been identified as a significant risk factors (32). It follows that the quality of life of these patients can be negatively influenced (33). In detail, a systematic review published by Lindner and colleagues (34) demonstrates how the socio-economic status and the aforementioned sociodemographic variables represent target data to be considered in the clinic with subjects suffering from T1DM. The results are particularly serious with reference to the possibilities of presenting adverse outcomes such as ketoacidosis and other forms of significant alteration. With particular reference to individual variables, age has been identified as a significant predictor of disease progression (25, 26) included among the early-life factors (35). According to Tatti and Pavandeep, gender is an undervalued variable for subjects with T1DM (36). Other studies highlighted the importance of considering this variables in order to produce a complete overview of the factors including the progress of the pathology (37, 38), illness duration and related possible complications (39). In line with these data, Morone (40) suggests the need to grasp these data and compare them with psychological variables in order to produce interventions aimed at risk reduction. Furthermore, in accordance with the review by Turin and Drobnič Radobuljac (41), etiology and management of the T1DM would be strongly influenced by the phenomena taken into consideration in this contribution, therefore of both a sociodemographic and psychological nature.

In this regard, recent research consistently demonstrates an elevated risk of psychological difficulties among individuals with T1DM, with heightened prevalence rates of anxiety, depression, eating disorders, and other psychiatric conditions (6, 42–56). Such psychopathology has been shown to adversely affect patients’ life trajectories and adherence to treatment regimens. In adolescence, a critical developmental period, Dalsgaard and colleagues (57) reported that the incidence of psychological issues reached 16%, highlighting the need for focused investigation in this age group. To inform effective interventions and reduce prevalence rates, further empirical studies are required to delineate the scope of these adversities within the T1DM population. Specifically, it is essential to identify and characterize the most prominent psychopathological presentations through rigorous research designs, thereby providing a sound evidence base for intervention development.

Although, to the best of our knowledge, much of the existing literature has concentrated on Type 2 Diabetes Mellitus (T2DM), psychopathological research on T1DM remains relatively underdeveloped. While a number of important studies have explored emerging psychological issues in individuals with T1DM, there remains a paucity of research examining the psychological mechanisms that contribute to the onset, maintenance, and exacerbation of the illness. Notably, psychopathology has been repeatedly identified as a key factor undermining self-management and adherence to medical treatments in individuals with T1DM (21, 41, 58–61). For example, Bădescu and colleagues (62) highlighted that depression could be up to three-times higher in participants suffering from T1DM and double for patients suffering from T2DM, in line with other studies evidencing its presence and common links and neurobiological dynamics (63–65). The association between anxiety and depression has been clearly detected and considered as negatively influencing disease management, glycemic control and adherence to treatments (66–68).

Eating disorders have been found to be present and lead to higher risk of complications directly related to poor glycemic control and due consequences (51, 69–76). The importance of maintaining consistent glycemic regulation and regular glucose monitoring is well established in the literature, particularly given the serious health consequences associated with poor metabolic management (77–80).

The role of obsession, defined as intrusive, unwanted thoughts, urges, or images that cause significant anxiety, has gained less attention in the field of T1DM than other psychopathological fields. While depression and anxiety have been widely studied over the past decades, obsessive-compulsive disorders (OCD) and related features remain relatively neglected. Although some studies have addressed obsessive-compulsive behaviors indirectly, only recently has research begun to engage more directly with this area (48, 81–83). Nonetheless, there remains a marked paucity of studies specifically addressing obsessive phenomena in individuals with T1DM. In contrast, emerging evidence has begun to document obsessive-compulsive features in relation to (84–86), yet equivalent investigations in T1DM populations are notably lacking. There is a clear need for greater empirical and clinical focus on the relevance of obsessive-compulsive symptoms in T1DM, particularly given their potential implications for psychological wellbeing and illness management.

The field of somatization represents a particularly relevant area of inquiry in the context of chronic illness. As observed in other medical conditions (87, 88), psychosomatic phenomena are often closely linked to underlying psychological processes. In the case of T1DM, emerging evidence suggests that psychological functioning plays a significant role in the onset, progression, and potential exacerbation of the disease (89–93). However, further investigation is needed to better understand the specific psychological mechanisms that contribute to the development of these factors. For instance, some recent studies highlighted this need with specific reference to some major concerns. According to Alazmi and colleagues’ systematic review (7), patients reported greater scores for depression, fear oh hypoglycemia and stress. Data is confirmed by other significant reviews and meta-analyses addressing stress, suicide risk, and somatization (49, 94–96).

One of the most important phenomena related to psychosomatics and psychopathology is alexithymia. Alexithymia can be defined as the difficulty to recognize, identify and describe affective phenomena such as emotions, feelings and mood (97–103). Alexithymia has been widely conceptualized and measured by a three factors model by Bagby et al. (1994), constituting difficulty identifying feelings, difficulty describing feelings and externally oriented thinking (15, 104–107).

The role of alexithymia in the context of chronic health conditions has been increasingly recognized and continues to attract scholarly attention (108–110). Within the diabetes literature, however, research has predominantly focused on T2DM (111, 112), with significantly less attention directed towards T1DM. This relative neglect has resulted in a limited understanding of the potential associations between alexithymia and psychological functioning in individuals with T1DM. Investigating the interplay between alexithymia and other psychological variables in this population thus represents a meaningful and timely area of inquiry. A study by Housiaux and colleagues (113) directly linked alexithymia to deteriorating glycemic control, while other research has demonstrated its adverse impact on disease management (114), glycemic control (115) and its potential role in predicting diabetes-related complications (116). Despite these findings, only a small number of studies have specifically explored the relationship between alexithymia and psychopathology within T1DM populations (23, 117–119), underscoring the need for further focused investigation.

A particularly robust association has been identified between alexithymia and intolerance of uncertainty (120–123). However, this relationship remains underexplored in the contexts of T1DM (23, 118) and longitudinal studies involving pathological groups and healthy controls (124). Intolerance of uncertainty is typically conceptualized as a dispositional psychological trait characterized by negative beliefs about uncertainty and its implications, often associated with heightened risk for a wide range of disruptions in several domains, such as clinical worry, emotional and behavioural responses (125–129). IU typically includes two main factors, respectively prospective and inhibitory anxiety. The psychopathological significance of this construct has garnered increasing interest across both clinical and non-clinical populations (120, 122, 130–134). In particular, Larking and colleagues (122) identified a significant link between alexithymia, intolerance of uncertainty, and somatization, while Abbate-Daga et al. (123) highlighted their combined relevance in the context of eating disorders. Similarly, Ozsivadjian and colleagues, as well as Brown and colleagues, demonstrated the importance of these constructs in internalizing and externalizing phenomena in individuals with neurodevelopmental disorders (120, 135).

Brown and colleagues (135) further substantiated these links through a systematic review and meta-analysis, showing that the interaction between alexithymia and intolerance of uncertainty may meaningfully impact health outcomes. Despite these findings, there is a notable absence of systematic reviews or meta-analyses specifically addressing this relationship within adolescent and emerging adult populations. Given the relevance of these constructs in various clinical domains, they have been incorporated into predictive models of general psychopathology (98, 136–140). These models have demonstrated significant explanatory power and continue to generate interest for future research, particularly in understanding the psychological mechanisms that influence chronic illness outcomes.

However, there remains a clear lack of research directly examining the interrelationships among alexithymia, intolerance of uncertainty, and psychopathological features in individuals with Type 1 Diabetes Mellitus (T1DM). The present study seeks to address this gap by investigating the associations, differences, and potential dependencies among these psychological constructs within a T1DM population.

### 1.1 Study hypotheses

Based on the emerged state of the art, we hypothesize that:

Hypothesis 1: There are significant correlations among sociodemographic variables, alexithymia, intolerance to uncertainty and psychopathological factors.

Hypothesis 2: Alexithymia and intolerance to uncertainty predict psychopathological factors.

Hypothesis 3: There are significant differences between age groups with reference to alexithymia, intolerance to uncertainty and psychopathological factors.

Hypothesis 4: There are significant differences between gender groups considering alexithymia, intolerance to uncertainty and psychopathological factors.

## 2 Materials and methods

### 2.1 Participants

Cross-sectional clinical research involving 150 participants aged between 11 and 18 years old (Mean: 14.09; SD: 2.37; 65.3% female) diagnosed with T1DM. All patients were recruited at the Pediatric Unit of the Ospedali Riuniti of Reggio Calabria (Italy; Director Dr. Domenico Minasi) routinely. The study was conducted from November 2023 to December 2024. All patients were under pharmacological treatment for T1DM and were diagnosed by an expert MD. The inclusion criteria were diagnosis of T1DM and evidence of appropriate medical treatment. The exclusion criteria were absence of comorbidity with other physical illnesses, absence of previously diagnosed psychopathological diseases, use of alcohol, drugs, or medications influencing cognitive functioning. All patients, including parents/tutors for minors, were informed about the anonymous nature of data. All patients fully completed the protocol. Each included subject and parents/tutors received and signed the written informed consent. Only 0.66% of subjects invited to participate in the study or their parents/tutors refused. No selection of subjects or subsampling based on further criteria was performed. The sample consists of data referring to all patients who were effectively involved in the study. This recruitment represents original psychological assessment performed in the clinical reality of reference.

### 2.2 Instruments

The present study was conducted using a protocol comprising a clinical interview, sociodemographic questionnaire and a set of validated psychometric instruments. Sociodemographic variables collected included participants’ age, gender, age at diagnosis, duration of illness, and educational attainment (measured in years of formal education).

#### 2.2.1 Self-administrated psychiatric scales for children and adolescents

Self-Administrated Psychiatric Scales for Children and Adolescents (SAFA) (141, 142) is a self-report clinical instrument validated in 2001 and developed by Cianchetti and Sannio Fratello (141). The instrument allows clinicians to build a complete report through principal scales and related subscales. The scales items are based on a 3-point Likert-type scale ranging from “not at all” to “entirely”. Each scale is composed of sub-scales related to psychopathological dynamics strictly linked to the main domain. Subscales of anxiety are for generalized, social, separation and school anxiety. Depression scale includes depressed mood, anhedonia, irritable mood, low self-esteem, insecurity, guilt and desperation. Obsessive-compulsive symptoms scale includes obsessive thoughts, compulsions/rituals, contamination, control and indecision. Psychogenic eating disorders scale includes bulimic behavior, anorexic behavior, body image and psychological aspects related to eating disorders. Somatic and hypochondria symptoms scale includes somatic and hypochondria symptoms.

The original validation provided for the following Cronbach’s alphas: Anxiety=0.887 for the nonclinical sample and 0.956 for the clinical sample (test–retest Pearson r:.913); Depression=0.909 for the nonclinical sample and 0.943 for the clinical sample (test–retest Pearson r:.881, highly significant); Obsession=0.916 for the nonclinical sample and 0.895 for the clinical sample (test–retest Pearson r: 0.820); Psychogenic eating disorders=0.814 for the nonclinical sample (test–retest Pearson r: 0.740, highly significant); Somatic symptoms and hypochondria = 0.876 for the nonclinical sample and 0.797 for the clinical sample (test-retest Pearson r: 0.567, highly significant).

#### 2.2.2 Toronto Alexithymia Scale (TAS-20)

TAS-20 (143) is a 20 items self-report scale based on a 5-point Likert-type scale ranging from “strongly disagree” to “strongly agree”. The scale is composed of three main factors - difficulty identifying feelings, difficulty describing feelings and externally oriented thinking. The instrument allows clinicians to assess alexithymia and related phenomena (see factors) through a valid and reliable widely recognized instrument. Thus, the use of TAS-20 in adolescent populations has been proved as effective (144–146). The validation of the original version of the scale produced the following Cronbach’s alpha indexes: 0.81 for the full scale; 0.78 for difficulty identifying feelings (DIF); 0.75 for difficulty describing feelings (DDF); 0.66 for externally oriented thinking (EOT). The Italian adaptation (147) of the scale provided for: 0.82 for the full scale, 0.79 for the first factor, 0.68 for the third factor and 0.54 for the last factor.

#### 2.2.3 Intolerance to Uncertainty Scale (IUS-12)

IUS-12 (148) is a self-report instrument assessing intolerance to uncertainty through 12 items based on a 5-point Likert scale ranging from “not at all characteristic of me” to “entirely characteristic of me”. The scale is composed of two main factors, respectively prospective and inhibitory anxiety. Its value has been demonstrated in different contests and samples, including adolescents (149–151). The original validation study demonstrated a good reliability (Cronbach’s alphas): 0.91 for the whole scale, 0.85 for both factors. The Italian validation (152) of the scale demonstrated good reliability for both full scale (0.80) and related factors (0.68 for prospective anxiety and 0.79 for inhibitory anxiety).

### 2.3 Ethics

The present study and all the related procedures were consistent with the Declaration of Helsinki (1964) and its amendments. The study was approved by the Ethical Committee “Comitato Etico Regionale Sezione Area Sud, Grande Ospedale Metropolitano Bianchi-Melacrino-Morelli” (Reggio Calabria), approval number: 19-2022 (27th April 2022).

### 2.4 Statistical analysis

For the statistical analyses categorial variables were expressed as number and percentage and numerical data through means and standard deviation. The Kolmogorov-Smirnov test was used to assess the normality of the distribution. After detecting the non-normality of some of the distributions, a non-parametric approach was chosen. Correlation analyses were performed using the Spearman Rho test. Generalized linear regressions were conducted to examine the relationship between clinical variables from a set of predictors (alexithymia and intolerance to uncertainty). Avoiding confounding effects, total score predictors analyses were performed separately from related factors. Statistical differences were studied using the Mann-Whitney test. A significance level of.05 was used for all analyses. A correction for multiple comparisons has not been applied as analyses were exploratory at this stage. Statistical analyses were performed using SPSS.25 for Windows package.

## 3 Results

The sequence of tables covers descriptive statistics, correlations, regressions and differential analyses.

**Table 1** includes the descriptive statistics expressed through mean and standard deviation for all study variables.

**Table 1:** Descriptive statistics for sociodemographic and clinical variables.

Variables	Mean	Standard deviation
Age	14.09	2.374
Education	8.93	2.463
Age of the diagnosis	7.95	3.495
Illness duration	6.140	3.745
TAS-20 Total score	53.440	12.337
TAS-20 Difficulty identifying feelings	18.133	6.720
TAS-20 Difficulty describing feelings	14.246	5.316
TAS-20 Externally oriented thinking	21.046	4.774
IUS-12 Total score	35.620	8.891
IUS-12 Prospective anxiety	22.313	5.349
IUS-12 Inhibitory anxiety	13.306	5.155
SAFA Anxiety-Total score	49.873	20.566
SAFA Anxiety- Generalized anxiety	13.160	5.993
SAFA Anxiety-Social anxiety	11.360	6.214
SAFA Anxiety-Separation anxiety	12.033	5.858
SAFA Anxiety-School anxiety	8.813	4.708
SAFA Depression-Total score	57.120	23.459
SAFA Depression-Depressed mood	7.675	4.197
SAFA Depression-Anhedonia	6.793	4.492
SAFA Depression-Irritable mood	7.0667	3.476
SAFA Depression-Low self-esteem	7.366	4.182
SAFA Depression-Insecurity	7.133	3.300
SAFA Depression-Guilt	6.846	3.943
SAFA Depression-Desperation	7.353	4.448
SAFA Obsessive-compulsive Symptoms-Total score	33.386	20.553
SAFA Obsessive-compulsive Symptoms-Obsessive thoughts	8.233	5.844
SAFA Obsessive-compulsive Symptoms-Compulsions/Rituals	8.146	5.521
SAFA Obsessive-compulsive Symptoms-Contamination	7.953	6.783
SAFA Obsessive-compulsive Symptoms-Control	4.240	3.490
SAFA Obsessive-compulsive Symptoms-Indecision	4.840	3.266
SAFA Psychogenic Eating Disorders-Total score	26.900	15.408
SAFA Psychogenic Eating Disorders-Bulimic behavior	6.606	5.680
SAFA Psychogenic Eating Disorders-Anorexic behavior	6.773	5.584
SAFA Psychogenic Eating Disorders-Body image	3.926	3.225
SAFA Psychogenic Eating Disorders-Psychological aspects	9.066	4.382
SAFA Somatic symptoms and hypochondria-Total score	22.020	15.058
SAFA Somatic symptoms and hypochondria-Somatic symptoms	5.880	4.011
SAFA Somatic symptoms and hypochondria-Hypochondria	3.420	3.008

*Hypothesis 1*

**Table 2** presents correlational analyses that included sociodemographic data and TAS-20 and IUS-12 scores. The first significant and negative correlation was age and externally oriented thinking. Thus, a significant and positive correlation was found with prospective anxiety. Considering education, the first significant and negative relationship regarded alexithymia (total score). A second significant and positive correlation involved externally oriented thinking. Age of the diagnosis produced two significant and positive relationships, involving alexithymia and difficulty describing feelings. Significant and negative correlations emerged among illness duration, alexithymia total score, difficulty identifying feelings and difficulty describing feelings. In line with this data, increasing age corresponded to decreased externally oriented thinking, representing a result consistent with maturation processes and the role of externally oriented thinking in subjects’ development. This tendency corresponded also to increasing education and related development of rational thinking. Alexithymia variables such as difficulty describing and difficulty identifying also assumed negative directions referring to illness duration and a positive direction with age of the diagnosis. The increasing age of diagnosis was linked to higher levels in difficulty describing feelings, while the relationship with illness duration was positive. The positive relationship between age of the diagnosis and alexithymia would represent a clear implication for possible interventions, while illness duration appeared to suggest the role of developmental processes influencing affective functioning of subjects.

**Table 2:** Correlation analyses among sociodemographic variables, alexithymia and intolerance to uncertainty.

Variables	Age	Education	Age of the diagnosis	Illness duration
TAS-20 Total score	-.150	-.180*	.161*	-.212**
TAS-20 Difficulty identifying feelings	-.041	-.063	.172*	-.162*
TAS-20 Difficulty describing feelings	-.013	-.035	.205*	-.185*
TAS-20 Externally oriented thinking	-.297**	.329**	-.106	-.049
IUS-12 Total score	.075	.050	.022	.060
IUS-12 Prospective anxiety	.165*	.146	-.020	.159
IUS-12 Inhibitory anxiety	-.043	-.062	.085	-.085

Bold values were significative values; *<0.05; **<0.001.

**Table 3** presents the correlational analyses performed between sociodemographic data and all clinical variables (SAFA scales). The first sociodemographic variable was age, presenting several significant correlations. Significant and negative correlations emerged with separation anxiety, school anxiety, depression (total scale score), anhedonia, low self-esteem guilt and hypochondria. A positive correlation emerged with reference to age and indecision (obsessive-compulsive scale). Considering education, significant and negative correlations emerged with separation anxiety, school anxiety and hypochondria, while a positive correlation regarded indecision. Illness duration presented several significant and negative correlations. The emerged significant results referred to anxiety (total score), social anxiety, separation anxiety, school anxiety, depression (total score), anhedonia, guilt and hypochondria. No significant relations emerged between age of the diagnosis and clinical variables. With reference to clinical implications related to emerging significant data, age assumed several negative directions towards psychopathological factors. Supporting these results, education represents a key variable for subject’s health status. The significant and negative correlations that emerged between psychopathological variables and illness duration suggest that maturation processes play a crucial role for the subjects.

**Table 3:** Correlation analyses among sociodemographic variables and psychopathology.

Variables	Age	Education	Age of the diagnosis	Illness duration
SAFA Anxiety-Total score	-.126	-.116	.131	-.205*
SAFA Anxiety- Generalized anxiety	.075	.071	.097	-.040
SAFA Anxiety-Social anxiety	-.110	-.097	.126	-.198*
SAFA Anxiety-Separation anxiety	-.252**	-.252**	.077	-.225**
SAFA Anxiety-School anxiety	-.176*	-.162*	.119	-.228**
SAFA Depression-Total score	-.192*	-.159	.066	-.175*
SAFA Depression-Depressed mood	-.072	-.058	.056	-.087
SAFA Depression-Anhedonia	-.186*	-.147	.089	-.196*
SAFA Depression-Irritable mood	-.058	-.049	.022	-.042
SAFA Depression-Low self-esteem	-.164*	-.148	.037	-.120
SAFA Depression-Insecurity	.010	.006	.030	-.010
SAFA Depression-Guilt	-.167*	-.139	.079	-.167*
SAFA Depression-Desperation	-.121	-.101	.093	-.158
SAFA Obsessive-compulsive Symptoms-Total score	.064	.085	.124	-.072
SAFA Obsessive-compulsive Symptoms-Obsessive thoughts	-.011	.000	.086	-.073
SAFA Obsessive-compulsive Symptoms-Compulsions/Rituals	.084	.105	.101	-.031
SAFA Obsessive-compulsive Symptoms-Contamination	.005	.043	.050	-.048
SAFA Obsessive-compulsive Symptoms-Control	.109	.112	.154	-.090
SAFA Obsessive-compulsive Symptoms-Indecision	.225**	.218**	.121	.044
SAFA Psychogenic Eating Disorders-Total score	-.067	-.041	.021	-.063
SAFA Psychogenic Eating Disorders-Bulimic behavior	-.142	-.112	-.029	-.077
SAFA Psychogenic Eating Disorders-Anorexic behavior	-.124	-.085	.027	-.105
SAFA Psychogenic Eating Disorders-Body image	-.089	-.080	-.039	-.011
SAFA Psychogenic Eating Disorders-Psychological aspects	.127	.130	.028	.062
SAFA Somatic symptoms and hypochondria-Total score	-.117	-.088	.046	-.110
SAFA Somatic symptoms and hypochondria-Somatic symptoms	-.063	-.025	.070	-.115
SAFA Somatic symptoms and hypochondria-Hypochondria	-.281**	-.239**	.038	-.211**

Bold values were significative values; *<0.05; **<0.001.

**Table 4** shows the correlation analyses between TAS-20 and IUS-12 variables. All emerged significant results were positive, including alexithymia total score, difficulty identifying feelings, difficulty describing feelings and all IUS-12 variables (total score, prospective and inhibitory anxiety. No significant correlations emerged with externally oriented thinking. The high number of significant and positive correlations emerged between alexithymia and intolerance to uncertainty represent an added value. The link regarding alexithymia and intolerance to uncertainty emerged as relevant and positive.

**Table 4:** Correlation analyses between alexithymia and intolerance to uncertainty variables.

Variables	TAS-20 total score	TAS-20 difficulty identifying feelings	TAS-20 difficulty describing feelings	TAS-20 externally oriented thinking
IUS-12 total score	.449**	.462**	.370**	.131
IUS-12 Prospective anxiety	.262**	.322**	.168*	.074
IUS-12 Inhibitory anxiety	.481**	.459**	.454**	.097

Bold values were significative values; *<0.05; **<0.001.

*Hypothesis 2*

**Table 5** reflects the generalized regression analyses between a set of predictors such as alexithymia and intolerance to uncertainty total scores and clinical variables (SAFA scales). The first significant and positive dependence emerged between intolerance to uncertainty and generalized anxiety. The second regarded intolerance to uncertainty and indecision, demonstrating the opposite direction of the phenomena. In line with this last relation, a significant and negative dependency emerged between intolerance to uncertainty and bulimic behavior, as well as for hypochondria. No significant relations emerged due to alexithymia predictor. The emerged significant dependencies highlight the predictive role of intolerance of uncertainty for anxiety, obsessive phenomena, eating and somatization symptoms.

**Table 5:** Generalized regression analysis among alexithymia and intolerance to uncertainty (predictors) and psychopathology.

Variables	TAS-20 total score		IUS-12 total score	
	B(CI: 95%)	*P*	B(CI: 95%)	*P*
SAFA Anxiety-Total score	.101 (-.165/.367)	.458	.257 (-.111/.662)	.171
SAFA Anxiety- Generalized anxiety	-.035 (-.042/.113)	.375	.138 (.032/.243)	.011*
SAFA Anxiety-Social anxiety	.028 (-.053/.108)	.501	-.017 (-.095/.129)	.767
SAFA Anxiety-Separation anxiety	.002 (-.085/.088)	.584	.060 (-.045/.165)	.262
SAFA Anxiety-School anxiety	.020 (-.041/.081)	.529	-.007 (-.078/.092)	.873
SAFA Depression-Total score	.094 (-.210/.398)	.544	-.060 (-.482/.363)	.782
SAFA Depression-Depressed mood	.033 (-.023/.088)	.245	-.018 (-.059/.095)	.639
SAFA Depression-Anhedonia	.003 (-.055/.062)	.912	-.029 (-.110/.051)	.476
SAFA Depression-Irritable mood	.022 (-.033/.076)	.437	-.011 (-.051/.074)	.726
SAFA Depression-Low self-esteem	.021 (-.041/.083)	.508	.015 (-.060/.090)	.962
SAFA Depression-Insecurity	.013 (-.030/.056)	.552	.024 (-.044/.092)	.489
SAFA Depression-Guilt	.010 (-.041/.061)	.697	-.027 (-.032/.801)	.371
SAFA Depression-Desperation	.027 (-.030/.085)	.858	-.012 (-.068/.092)	.764
SAFA Obsessive-compulsive Symptoms-Total score	-.003 (-.264/.269)	.983	.019 (-.351/.389)	.919
SAFA Obsessive-compulsive Symptoms-Obsessive thoughts	.015 (-.061/.091)	.702	.027 (-.078/.132)	.619
SAFA Obsessive-compulsive Symptoms-Compulsions/Rituals	-.002 (-.074/.069)	.951	.015 (-.084/.114)	.765
SAFA Obsessive-compulsive Symptoms-Contamination	.021 (-.109/.067)	.636	-.079 (-.201/.046)	.201
SAFA Obsessive-compulsive Symptoms-Control	.001 (-.044/.047)	.953	-.001 (-.063/.063)	.997
SAFA Obsessive-compulsive Symptoms-Indecision	.009 (-.033/.051)	.676	-.054 (-.004/.112)	.043*
SAFA Psychogenic Eating Disorders-Total score	.063 (-.136/.263)	.535	-.089 (-.365/.188)	.531
SAFA Psychogenic Eating Disorders-Bulimic behavior	.005 (-.079/.068)	.889	-.093 (-.194/.008)	.045*
SAFA Psychogenic Eating Disorders-Anorexic behavior	-.008 (-.080/.065)	.832	-.088 (-.187/.012)	.085
SAFA Psychogenic Eating Disorders-Body image	.019 (-.023/.060)	.380	-.003 (-.061/.055)	.913
SAFA Psychogenic Eating Disorders-Psychological aspects	.054 (-.002/.110)	.061	.043 (-.036/.122)	.284
SAFA Somatic symptoms and hypochondria-Total score	.051 (-.144/.246)	.609	-.090 (-.391/.180)	.513
SAFA Somatic symptoms and hypochondria-Somatic symptoms	.005 (-.047/.057)	.843	-.033 (-.105/.039)	.364
SAFA Somatic symptoms and hypochondria-Hypochondria	.004 (-.035/-.043)	.840	-.048 (-.102/.069)	.041*

Bold values were significative values; *<0.05.

**Table 6** reports the generalized regression analyses between alexithymia and intolerance to uncertainty factors (predictors) and clinical variables. Externally oriented thinking predicted higher levels of depression, anhedonia, irritable mood, low self-esteem, obsessive thoughts and body image issues. Prospective anxiety predicted higher levels of generalized anxiety and psychological aspects related to eating disorders. No significant dependencies emerged with reference to the predictors difficulty identifying, describing feelings and inhibitory anxiety. The predictive role of alexithymia on psychopathological phenomena appears crucial. Confirming the previous analyses, prospective anxiety predicted some relevant pathological domains.

**Table 6:** Generalized regression analysis among alexithymia and intolerance to uncertainty (predictors) and psychopathology.

Variables	TAS-20 DIF		TAS-20 DDF		TAS-20 EOT		IUS-12 PA		IUS-12 IA	
	B(CI: 95%)	*P*	B(CI: 95%)	*P*	B(CI: 95%)	*P*	B(CI: 95%)	*P*	B(CI: 95%)	*p*
SAFA Anxiety-Total score	.017 (-.628/.663)	.958	.019 (-.799/.837)	.964	.458 (-.231/1.147)	.193	.470 (-.270/1.147)	.173	.033 (-.670/.735)	.927
SAFA Anxiety- Generalized anxiety	-.049 (-.237/.138)	.607	.084 (-.153/.322)	.487	.149 (-.051/.350)	.143	.232 (.039/.426)	.019*	.038 (-.163/.239)	.711
SAFA Anxiety-Social anxiety	.098 (-.097/.293)	.326	-.097 (-.344/.150)	.443	.086 (-.123/.294)	.420	.123 (-.082/.329)	.238	-.095 (-.308/.118)	.381
SAFA Anxiety-Separation anxiety	-.013 (-.197/.171)	.892	.012 (-.221/.245)	.919	.128 (-.069/.324)	.203	.027 (-.166/.221)	.783	.095 (-.106/.296)	.355
SAFA Anxiety-School anxiety	-.024 (-.172/.123)	.745	.030 (-.157/.217)	.752	.107 (-.051/.264)	.184	.051 (.207/.414)	.520	-.040 (-.202/.122)	.630
SAFA Depression-Total score	.023 (-.712/.758)	.951	-.084 (-1.016/.847)	.859	.612 (-.172/1.397)	.049*	.076 (-.702/.854)	.848	-.202 (-1.009/.605)	.624
SAFA Depression-Depressed mood	-.007 (-.141/.126)	.913	.032 (-.137/.201)	.713	.137 (-.006/.279)	.060	.025 (-.116/.167)	.725	.011 (-.136/.158)	.883
SAFA Depression-Anhedonia	-.001 (-.143/.140)	.986	-.012 (-.191/.168)	.897	.041 (-.110/.192)	.025*	-.032 (-.181/.117)	.675	-.027 (-.181/.128)	.734
SAFA Depression-Irritable mood	-.014 (-.122/.094)	.795	.009 (-.127/.146)	.893	.136 (.021/.251)	.020*	.038 (-.077/.153)	.517	-.017 (-.137/.102)	.779
SAFA Depression-Low self-esteem	.028 (-.103/.158)	.678	-.051 (-.216/.114)	.547	.144 (.005/.283)	.042*	.055 (-.084/.193)	.437	-.027 (-.170/.117)	.716
SAFA Depression-Insecurity	-.058 (-.161/.045)	.272	.067 (-.063/.197)	.314	.086 (-.024/.196)	.124	.075 (-.034/.184)	.178	-.023 (-.136/.090)	.686
SAFA Depression-Guilt	-.019 (-.142/.105)	.767	.006 (-.151/.163)	.937	.089 (-.043/.221)	.187	.004 (-.126/.135)	.948	-.067 (-.202/.069)	.335
SAFA Depression-Desperation	.004 (-.136/.143)	.960	.015 (-.161/.192)	.864	.109 (-.039/.258)	.150	-.089 (-.199/.022)	.115	-.033 (-.148/.081)	.569
SAFA Obsessive-compulsive Symptoms-Total score	-.087 (-.734/.560)	.791	-.038 (-.858/.782)	.928	.303 (-.388/.993)	.390	.359 (-.320/1.038)	.300	-.339 (-1.043/.366)	.346
SAFA Obsessive-compulsive Symptoms-Obsessive thoughts	-.001 (-.183/.181)	.991	-.059 (-.290/.172)	.616	.197 (.003/.391)	.047*	.141 (-.052/.333)	.152	-.093 (-.293/.106)	.359
SAFA Obsessive-compulsive Symptoms-Compulsions/Rituals	-.050 (-.223/.124)	.574	.001 (-.219/.220)	.996	.110 (-.075/.295)	.244	.117 (-.065/.299)	.208	-.092 (-.281/.097)	.340
SAFA Obsessive-compulsive Symptoms-Contamination	.051 (-.163/.264)	.641	-.104 (-.375/.167)	.451	-.045 (-.273/.183)	.702	-.018 (-.242/.205)	.873	-.144 (-.376/.088)	.225
SAFA Obsessive-compulsive Symptoms-Control	-.052 (-.162/.057)	.349	.092 (-.047/.231)	.194	-.040 (-.157/.077)	.503	.031 (-.085/.147)	.600	-.033 (-.153/.087)	.592
SAFA Obsessive-compulsive Symptoms-Indecision	-.034 (-.136/.068)	.516	.026 (-.103/.156)	.690	.083 (-.026/.192)	.135	.089 (-.018/.196)	.103	.017 (-.094/.128)	.769
SAFA Psychogenic Eating Disorders-Total score	-.168 (-.652/.316)	.497	.304 (-.309/.918)	.331	.171 (-.346/.687)	.518	.103 (-.406/.612)	.691	-.290 (-.819/.238)	.282
SAFA Psychogenic Eating Disorders-Bulimic behavior	-.051 (-.230/.128)	.577	.038 (-.189/.265)	.740	.023 (-.168/.214)	.811	-.117 (-.303/.069)	.218	-.067 (-.261/.126)	.496
SAFA Psychogenic Eating Disorders-Anorexic behavior	-.024 (-.201/.152)	.786	-.008 (-.231/.216)	.946	.030 (-.158/.218)	.752	-.053 (-.236/.131)	.574	-.124 (-.315/.066)	.201
SAFA Psychogenic Eating Disorders-Body image	-.070 (-.170/.030)	.169	.087 (-.039/.213)	.176	.109 (.003/.215)	.045*	.003 (-.104/.110)	.962	-.009 (-.120/.102)	.868
SAFA Psychogenic Eating Disorders-Psychological aspects	-.050 (-.185/.086)	.470	.156 (-.016/.327)	.075	.117 (-.027/.262)	.112	.184 (.042/.326)	.011*	-.106 (-.253/.042)	.161
SAFA Somatic symptoms and hypochondria-Total score	.038 (-.435/.511)	.875	-.079 (-.678/.520)	.797	.330 (-.175/.834)	.200	.067 (-.431/.565)	.793	-.256 (-.772/.261)	.332
SAFA Somatic symptoms and hypochondria-Somatic symptoms	.012 (-.115/.138)	.859	.007 (-.153/.168)	.930	-.018 (-.153/.117)	.798	-.001 (-.133/.132)	.992	-.068 (-.205/.070)	.335
SAFA Somatic symptoms and hypochondria-Hypochondria	.012 (-.082/.106)	.801	-.038 (-.158/.082)	.534	.064 (-.036/.165)	.210	-.031 (-.130/.068)	.536	-.066 (-.168/.037)	.210

Bold values were significative values; *<0.05; TAS DIF, Difficulty identifying feelings; TAS DDF, Difficulty describing feelings; TAS EOT, Externally-oriented thinking. IUS-12 PA, Prospective anxiety; IUS-12 IA, Inhibitory anxiety.

*Hypothesis 3*

**Table 7** includes the differential analyses performed with reference to two age groups (11–14 and 15-18). The first significant difference was found considering externally oriented thinking with higher scores in younger participants. The second significant difference regarded separation anxiety, in line with the previous one. Higher scores of anhedonia were found in younger participants. Indecision appeared significantly higher in older participants, as well as psychological aspects related to eating disorders. Hypochondria appeared significantly higher in younger participants. Results lead to clinical implications. Externally oriented thinking refers more to younger participants, as well as separation anxiety. In these terms, increasing age corresponds to diminishing rate of the considered phenomena. Hypochondria and anhedonia represent significant phenomena related to somatization and depressive symptomatology.

**Table 7:** Comparison between age groups.

Variables	11–14 years old (*N* = 86) (Mean ± SD)	15–18 years old (*N=66*) (Mean ± SD)	*P*-value
TAS-20 Total score	54.357 ± 12.204	52.272 ± 12.499	.270
TAS-20 Difficulty identifying feelings	18.178 ± 6.438	18.075 ± 7.112	.955
TAS-20 Difficulty describing feelings	14.166 ± 5.459	14.348 ± 5.169	.976
TAS-20 Externally oriented thinking	21.988 ± 4.405	19.848 ± 4.986	.008*
IUS-12 Total score	34.988 ± 8.407	36.424 ± 9.475	.437
IUS-12 Prospective anxiety	21.523 ± 5.047	23.318 ± 5.588	.058
IUS-12 Inhibitory anxiety	13.464 ± 4.808	13.106 ± 5.597	.491
SAFA Anxiety-Total score	51.309 ± 21.886	48.045 ± 18.758	.368
SAFA Anxiety- Generalized anxiety	12.464 ± 6.202	14.045 ± 5.638	.170
SAFA Anxiety-Social anxiety	11.773 ± 6.462	10.833 ± 5.890	.454
SAFA Anxiety-Separation anxiety	13.083 ± 6.133	10.697 ± 5.235	.016*
SAFA Anxiety-School anxiety	9.178 ± 4.820	8.348 ± 4.555	.246
SAFA Depression-Total score	60.309 ± 25.039	53.060 ± 20.763	.082
SAFA Depression-Depressed mood	8.142 ± 4.618	7.333 ± 3.792	.275
SAFA Depression-Anhedonia	7.559± 4.820	5.818± 3.854	.047*
SAFA Depression-Irritable mood	7.166 ± 3.737	6.939 ± 3.137	.591
SAFA Depression-Low self-esteem	7.833 ± 4.565	6.772± 3.585	.167
SAFA Depression-Insecurity	7.011± 3.370	7.287 ± 3.228	.500
SAFA Depression-Guilt	7.333 ± 4.093	6.227± 3.682	.097
SAFA Depression-Desperation	7.761 ± 4.853	6.833 ± 3.845	.236
SAFA Obsessive-compulsive Symptoms-Total score	31.5 ± 21.617	35.787 ± 19.004	.201
SAFA Obsessive-compulsive Symptoms-Obsessive thoughts	8± 5.89261	8.530± 5.813	.525
SAFA Obsessive-compulsive Symptoms-Compulsions/Rituals	7.476 ± 5.528	9± 5.434	.121
SAFA Obsessive-compulsive Symptoms-Contamination	8.214 ± 7.259	7.621 ± 6.163	.935
SAFA Obsessive-compulsive Symptoms-Control	3.821± 3.550	4.772 ± 3.364	.075
SAFA Obsessive-compulsive Symptoms-Indecision	4.035 ± 3.125	5.863 ± 3.176	.001*
SAFA Psychogenic Eating Disorders-Total score	26.726 ± 16.736	27.121 ± 13.656	.944
SAFA Psychogenic Eating Disorders-Bulimic behavior	7.083 ± 6.096	6 ± 5.083	.461
SAFA Psychogenic Eating Disorders-Anorexic behavior	7.285 ± 6.094	6.121 ± 4.824	.304
SAFA Psychogenic Eating Disorders-Body image	3.976 ± 3.267	3.863 ± 3.195	.853
SAFA Psychogenic Eating Disorders-Psychological aspects	8.381 ± 4.387	9.939 ± 4.249	.036*
SAFA Somatic symptoms and hypochondria-Total score	22.738 ± 15.6483	21.106 ± 14.337	.548
SAFA Somatic symptoms and hypochondria-Somatic symptoms	5.904 ± 4.250	5.848 ± 3.717	.995
SAFA Somatic symptoms and hypochondria-Hypochondria	3.976 ± 3.013	2.712 ± 2.870	.008*

Bold values were significative values; *<0.05.

*Hypothesis 4*

**Table 8** reports differential analyses between male and female groups. The only significant difference emerged in relation to difficulty identifying feelings, with higher scores in female participants. No other significant differences were found between male and female groups.

**Table 8:** Comparison between gender groups.

Variables	Male (*N=*52) (Mean ± SD)	Female (*N=*98) (Mean ± SD)	*P*-value
TAS-20 Total score	52.365 ± 12.569	54.010 ± 12.238	.265
TAS-20 Difficulty identifying feelings	16.653 ± 7.262	18.918 ± 6.312	.042*
TAS-20 Difficulty describing feelings	13.846 ± 5.026	14.459 ± 5.477	.545
TAS-20 Externally oriented thinking	21.826 ± 4.162	20.632 ± 5.040	.166
IUS-12 Total score	35.519 ± 7.882	35.673 ± 9.421	.782
IUS-12 Prospective anxiety	22.788 ± 4.525	22.061 ± 5.745	.825
IUS-12 Inhibitory anxiety	12.730 ± 5.27709	13.612 ± 5.090	.364
SAFA Anxiety-Total score	52.307 ± 23.972	48.581 ± 18.512	.424
SAFA Anxiety- Generalized anxiety	13.250 ± 6.239	13.112 ± 5.890	.785
SAFA Anxiety-Social anxiety	11.942 ± 7.298	11.051 ± 5.571	.541
SAFA Anxiety-Separation anxiety	12.788 ± 6.493	11.632 ± 5.485	.306
SAFA Anxiety-School anxiety	9.211 ± 4.754	8.602 ± 4.69424	.473
SAFA Depression-Total score	61.788 ± 26.919	54.642 ± 21.131	.081
SAFA Depression-Depressed mood	8.403 ± 5.014	7.459 ± 3.821	.204
SAFA Depression-Anhedonia	7.423 ± 4.975	6.459 ± 4.201	.326
SAFA Depression-Irritable mood	7.576 ± 3.268	6.795 ± 3.569	.191
SAFA Depression-Low self-esteem	7.788 ± 4.971	7.142 ± 3.705	.371
SAFA Depression-Insecurity	7.519 ± 3.380	6.928 ± 3.256	.379
SAFA Depression-Guilt	7.557 ± 4.344	6.469 ± 3.681	.102
SAFA Depression-Desperation	7.788 ± 5.289	7.122 ± 3.940	.331
SAFA Obsessive-compulsive Symptoms-Total score	34.615 ± 23.608	32.734 ± 18.832	.684
SAFA Obsessive-compulsive Symptoms-Obsessive thoughts	8.519 ± 6.551	8.081 ± 5.461	.743
SAFA Obsessive-compulsive Symptoms-Compulsions/Rituals	8.326 ± 5.724	8.051 ± 5.438	.688
SAFA Obsessive-compulsive Symptoms-Contamination	8.346 ± 7.205	7.744 ± 6.577	.689
SAFA Obsessive-compulsive Symptoms-Control	4.538 ± 3.472	4.081 ± 3.507	.470
SAFA Obsessive-compulsive Symptoms-Indecision	4.884 ± 2.941	4.816 ± 3.441	.874
SAFA Psychogenic Eating Disorders-Total score	28.596 ± 17.157	26 ± 14.407	.303
SAFA Psychogenic Eating Disorders-Bulimic behavior	7.538 ± 5.758	6.112 ± 5.605	.107
SAFA Psychogenic Eating Disorders-Anorexic behavior	7.211 ± 6.095	6.540 ± 5.311	.593
SAFA Psychogenic Eating Disorders-Body image	4.173 ± 3.364	3.795 ± 3.158	.369
SAFA Psychogenic Eating Disorders-Psychological aspects	9 ± 4.601	9.102 ± 4.284	.972
SAFA Somatic symptoms and hypochondria-Total score	23.480 ± 17.206	21.244 ± 13.816	.449
SAFA Somatic symptoms and hypochondria-Somatic symptoms	6.711 ± 4.499	5.438 ± 3.675	.106
SAFA Somatic symptoms and hypochondria-Hypochondria	3.961 ± 3.093	3.132 ± 2.937	.104

Bold values were significative values; *<0.05.

## 4 Discussion

The present study investigated the relationships among sociodemographic variables, alexithymia, intolerance of uncertainty, and psychopathological factors in a sample of young individuals with T1DM. Guided by four hypotheses, the study first examined whether significant correlations existed among these variables (Hypothesis 1). It then explored the predictive value of alexithymia and intolerance of uncertainty on psychopathological features (Hypothesis 2). To further assess group-level differences, the study conducted comparative analyses to evaluate whether age groups differed significantly in levels of alexithymia, intolerance of uncertainty, and psychopathological symptoms (Hypothesis 3), and whether similar differences emerged across gender groups (Hypothesis 4). Through this multi-level analytic approach, the study aimed to clarify the interplay of psychological and demographic factors in the mental health profiles of individuals with T1DM.

The first hypothesis addressed potential correlations among sociodemographic variables, alexithymia, intolerance of uncertainty and psychopathological factors. In relation to alexithymia, both educational attainment and illness duration were negatively associated with total alexithymia scores, suggesting that higher levels of education and longer illness duration may be linked to lower levels of alexithymic traits. These findings are consistent with prior literature (153–156). Specifically, illness duration was inversely related to total alexithymia scores, as well as to the subscales assessing difficulty identifying feelings and difficulty describing feelings. While several studies have explored the association between alexithymia and physical illness, there remains a lack of clear evidence specifically examining its relationship with illness duration.

Externally oriented thinking demonstrated a positive correlation with education and a negative correlation with age, a pattern aligned with previous research on developmental changes in emotional processing (157, 158), while age was positively associated with prospective anxiety on the Intolerance of Uncertainty Scale (IUS-12), suggesting a possible decline in psychological flexibility and overall mental health with age, as supported by studies such as Okayama and colleagues (159) and others (149, 160).

Conversely, increasing age was linked to lower levels of separation anxiety, school-related anxiety, depression, anhedonia, low self-esteem, guilt and hypochondriasis. These findings are consistent with evidence indicating a general reduction in anxiety and depressive symptoms across adolescence, likely due to neurobiological maturation (161–163). However, as previously noted, the presence of a chronic condition such as T1DM constitutes a significant risk factor for psychopathological expression (164). Therefore, timely psychological intervention should be considered a crucial component of care following the identification of psychological distress.

Higher levels of education were associated with lower levels of separation anxiety, school-related anxiety, and hypochondriasis. Additionally, indecisiveness showed a significant positive association with both age and education. While the protective role of education in mitigating risk for psychopathology is well established in the general population (165–168), limited empirical evidence exists regarding its specific impact in individuals with T1DM. These findings suggest that educational attainment may serve as a potential buffer against certain psychological issues in this clinical group, although considering the exploratory nature of the study, further research is needed to clarify the mechanisms underlying this relationship.

A particularly noteworthy finding was the set of significant negative correlations between illness duration and a range of psychopathological variables. Specifically, longer illness duration was significantly associated with lower levels of general anxiety, social anxiety, separation anxiety, school-related anxiety, depression, anhedonia, guilt, and hypochondriasis. These results may reflect a possible psychological adjustment process to living with a chronic illness over time, a phenomenon supported by previous research (169–171). However, it is important to note that such adaptive processes do not negate the need for appropriate psychological support. Even when indicators of distress decrease with time, structured psychological interventions remain essential to promote long-term well-being and adaptive functioning in individuals with T1DM.

The association between alexithymia and intolerance of uncertainty was clearly supported by the present findings, with all significant correlations being positive. These results are consistent with recent theoretical developments and empirical trends, further reinforcing the relevance of these constructs in health psychology. The interplay between alexithymia and intolerance of uncertainty is particularly noteworthy given their established influence on health-related outcomes. However, as previously noted, these constructs have rarely been examined in tandem within the context of T1DM. While existing literature has explored alexithymia and intolerance of uncertainty, primarily in isolation, in relation to T2DM, only a limited number of studies have included T1DM populations (113–115, 119, 124, 172, 173). This gap is especially evident with respect to intolerance of uncertainty, which remains under-investigated in T1DM and other chronic health conditions.

The second hypothesis focused on the predictive role of alexithymia and intolerance of uncertainty in relation to psychopathological factors. The link between anxiety and intolerance of uncertainty is well established (174, 175), and a recent review by Sternheim and colleagues (176) further underscored intolerance of uncertainty as a risk factor for the development of eating disorders. Although comprehensive reviews by Kesby and colleagues (177) and Brown and colleagues (135) have examined this relationship in broader contexts, studies explicitly addressing these associations in T1DM are still lacking. Of particular relevance, Brown and colleagues (135) reported a connection between intolerance of uncertainty and bulimia, highlighting associations with harm avoidance and depressive symptoms. These findings point to the potential clinical utility of targeting intolerance of uncertainty in psychological interventions, including for populations with chronic conditions such as T1DM.

Emotional regulation, alexithymia’s feeling identification and description have been found associated with orthorexic tendencies (178), diabetes management and glycemic control issues and presence of eating disorders in participants suffering from T1DM (23, 114, 118, 179). Accordingly, the present findings align with existing literature, reinforcing the relevance of alexithymic traits in the psychological and clinical profiles of individuals with T1DM. Furthermore, consistent with the hypothesized predictive role of intolerance of uncertainty, prospective anxiety was found to possibly predict elevated levels of generalized anxiety and hypochondriasis. These results support and extend previous findings, further emphasizing the importance of targeting intolerance of uncertainty and alexithymic traits in interventions aimed at improving mental health and self-management outcomes in this population.

The third hypothesis explored potential age-related differences in alexithymia and associated psychopathological variables. A significant difference was found in externally oriented thinking, with younger participants exhibiting higher levels of this cognitive style. Additionally, younger individuals reported greater levels of separation anxiety, anhedonia, and hypochondriasis. In contrast, older participants demonstrated higher levels of indecision and psychological difficulties related to eating disorders.

Although previous research has reported comparable levels of alexithymia across paediatric and adult populations with T1DM (155), and few studies have specifically examined age-related variations in emotional recognition (180, 181), the present findings underscore the developmental sensitivity of externally oriented thinking. This result is consistent with a recent meta-analysis highlighting the developmental dependence of externally oriented thinking, particularly during adolescence and early adulthood (182). Regarding hypochondriasis, earlier studies have typically reported no significant age-related differences (183), with only limited evidence pointing to developmental trends in somatization and health anxiety (184). However, it is important to note that most of this literature is based on general or psychiatric populations rather than individuals with chronic medical conditions such as T1DM. The present study contributes novel insights by identifying age-specific psychological patterns within this clinical group, underscoring the importance of developmental considerations in both research and intervention design.

The final hypothesis examined gender differences in alexithymia, intolerance of uncertainty, and psychopathological variables. The only statistically significant difference emerged in the domain of alexithymia, specifically in difficulty identifying feelings, with female participants reporting higher levels. This finding is consistent with a recent meta-analysis by Mendia and colleagues (182), which highlighted gender-related differences in alexithymia—particularly during adolescence—with females more likely to report greater difficulty identifying emotions. These gender differences appeared to diminish in young adulthood, underscoring the developmental variability of emotional processing. Thus, this interesting result should be considered in the light of the influence provided by socialization effects and response biases. Even in this case, more studies are needed. Furthermore, consistent with the age-related analyses, younger participants also reported higher scores on the difficulty identifying feelings subscale, suggesting a broader trend linked to developmental stage.

Overall, the four hypotheses facilitated the identification of key psychological characteristics and group-level differences within individuals living with T1DM, contributing to a deeper understanding of an underexplored area. Despite the emergence of several significant and clinically relevant findings, the cross-sectional design of the present study limits the ability to draw causal inferences. Single hospital unit, absence of a control group and recruitment performed through routine visits represent another limit for the study. Nevertheless, these limitations highlight the need for longitudinal and experimental research to further investigate the developmental trajectories and psychological mechanisms implicated in T1DM. Building on the current results, future studies should aim to develop targeted interventions to mitigate psychopathological symptoms and related emotional difficulties in this population (185). Moreover, it is of fundamental importance that these interventions are realistic and sustainable for regular implementation by clinicians.

## 5 Conclusions

The findings of this study underscore the importance of alexithymia, intolerance of uncertainty and psychopathological factors in the context of chronic illness, with a specific focus on T1DM. By examining these variables in a sample of young individuals, the study sought to illuminate their interrelationships and identify developmental and gender-based differences. Consistent patterns emerged indicating the relevance of alexithymia and intolerance of uncertainty in the psychological profile of T1DM patients, with significant associations also observed with psychopathological symptoms. Thus, intolerance to uncertainty predicted anxiety and obsessive phenomena, while alexithymia showed fewer predictive associations. The significant associations observed offer valuable insights for the development of targeted, evidence-based psychological interventions. Given the potential health risks associated with unaddressed psychological distress in T1DM, the continued neglect of these factors in clinical practice and research cannot be justified. Closing this gap is critical to advancing both the scientific understanding and holistic care of individuals with T1DM.

## Data Availability

The raw data supporting the conclusions of this article will be made available by the authors, without undue reservation.
